# Demonstrations of Anatomy; Being a Guide to the Dissection of the Human Body

**Published:** 1841-01

**Authors:** 


Art. III.—*Demonstrations of Anatomy; being a Guide to the Dissection of the Human Body.* By George Viner Ellis, one of the Demonstrators of Anatomy in University College.—*London,* 1840. 8vo, pp. 620.

This is a novel and appropriate title to a book intended expressly as a guide in the dissecting-room. But after the appearance of so many "guides" and "dissectors," we had hardly hoped to find anything new in the plan or execution of the one now before us. In this we have been agreeably mistaken. In the present volume the information is solid and practical; there are none of those panderings to idleness in the shape of cuts and diagrams, which, however they may be tolerated or even be serviceable in works of mere descriptive anatomy, we think are quite unnecessary in a demonstrative work like the present, which is intended to be used with the objects described before the eyes of the student. We cannot better or more concisely indicate the plan of Mr. Ellis's book, as compared with that of the "dissectors" usually employed, than by quoting his own words:

"Anatomy may be studied in two different methods, the one demonstrative and the other descriptive: the former treats of the parts only as they are exposed in dissection, and of their relative position; the latter gives minute details of objects unseen, describing the different structures without reference to the order in which they appear, and has been very generally employed in the numerous works of instruction in practical anatomy; but every one acquainted with the difficulties to be overcome by the young dissector will readily admit that this method is insufficient as a guide to his progress." (Preface.)

The difficulties alluded to are almost or entirely obviated in the plan of the present work.

"The great divisions of the body, head, neck, thorax, bulk, extremities, and abdomen have been placed in different sections, and the dissection of each has been conducted by the employment of successive stages, determined either by certain apparently natural limits, or by those most convenient in practice; by means of full directions for the performance of the different steps; by describing at one time only so much of a part as is visible, and by noting each as it appears." (Preface.)

We would refer, as instances of the general utility to the student of the plan of this work, to the dissections of the portia dura and of the orbit, or of the back, in which the vessels and nerves are described as well as muscles, and the method to be followed in exposing each part is laid down with such care and precision as must greatly facilitate the progress of the dissector. This attention is not confined to one set of structures or portion of the body. Even in the dissection of the abdomen, where the student is often much at a loss how to proceed, owing to the little attention that has been paid in most works of anatomy to the order in which particular parts should be studied, and the stages of the dissection in which they may be best seen, the author has bestowed the same care and attention as on other regions. As an example of the precision with which the dissections for these "demonstrations" have been followed out, we may instance that of the deep temporal branches of the fifth cranial nerve. It is usually stated in most anatomical works that several cutaneous filaments perforate the fascia, ramify in the integuments, and join with the portia dura: but the author shows that the branches that perforate the fascia to join with the portia dura nerve are derived from the orbital branch of the superior maxillary nerve, or second division of the fifth. (p. 99.) Again, the nerve of Wrisberg is correctly described as arising from the brachial plexus, and its junction with the intercosto-humeral is alluded to. (p. 318.)

We are much pleased to observe that the author has paid rather more attention than is usual in most English works to the dissection of the nervous system, not so much perhaps in the addition of new facts as in —what in our estimation is almost equally meritorious — carefully examining what has been done by others, and dissecting to verify the same. Thus at page 569 he has described the *accessory of the obturator* of Schmidt, which is so frequently present, but is commonly passed over in silence.

In the dissection of the brain and spinal cord, the author has availed himself of the valuable manuscript notes of Dr. Sharpey, and throughout his whole work has carefully added whatever new anatomical fact has been recently established or has been overlooked. We think that the study of relative and surgical anatomy will be greatly assisted by the descriptions given of the different regions, as in the triangles of the neck, axilla, bend of the elbow, &c.; in a word, we think Mr. Ellis's "Demonstrations" are in every way fitted for the purpose for which they are intended, and we therefore strongly recommend the work to the notice of every member of the profession. We are convinced that it will quickly become the general text-book of every working student in anatomy.

